# Therapeutic options for treatment of human papillomavirus-associated cancers - novel immunologic vaccines: ADXS11–001

**DOI:** 10.1186/s40661-017-0047-8

**Published:** 2017-07-14

**Authors:** Brett Miles, Howard P. Safran, Bradley J. Monk

**Affiliations:** 1 Department of Otolaryngology, Icahn School of Medicine at Mount Sinai, New York, NY USA; 2 Brown University Oncology Research Group, Providence, RI USA; 3 Division of Gynecologic Oncology, Arizona Oncology (US Oncology Network), University of Arizona College of Medicine, Creighton University School of Medicine at St. Joseph’s Hospital, 2222 E. Highland Ave, Suite 400, Phoenix, AZ 85016 USA

**Keywords:** ADXS11–001, AXAL, Human papillomavirus, HPV-positive cancers, Clinical trials

## Abstract

Survival of patients with advanced, recurrent, or metastatic human papillomavirus (HPV)-associated cancer is suboptimal despite the availability of various treatment modalities. The recently developed bacterial vector *Listeria monocytogenes* (*Lm*) activates innate and adaptive immune responses and is expected to offer immunologic advantages. Axalimogene filolisbac (AXAL or ADXS11–001) is a novel immunotherapeutic based on the live, irreversibly attenuated *Lm* fused to the nonhemolytic fragment of listeriolysin O (*Lm*-LLO) and secretes the *Lm*-LLO-HPV E7 fusion protein targeting HPV-positive tumors. Herein are reported the development and recent results of various clinical trials in patients with HPV-associated cervical, head and neck, and anal cancers.

## Introduction

Persistent human papillomavirus (HPV) infection is currently acknowledged as a direct cause of cervical, anogenital, and oropharyngeal cancers [1], and has been estimated to account for more than 5% of all cancers globally [2]. More than half of all cancers attributable to infection worldwide are caused by HPV (Table 1) [3]. Moreover, cervical cancer was the first type of cancer officially recognized by the World Health Organization to be attributable to a viral infection.

**Table 1:** Estimated number of HPV-attributable new cancer cases, by anatomic site and gender

Cancer site	Number of new cases	Number of cases attributable to HPV	Attributable fraction, %	Number of cases attributable to HPV by gender	
				Male	Female
Cervix uteri	528,000	501,600	95	-	528,000
Anus	40,000	35,000	88	17,000	18,000
Vagina and vulva	49,000	20,000	41	-	20,000
Penis	26,000	13,000	51	13,000	-
Oropharynx	96,000	29,000	31	24,000	6000
Oral cavity and larynx	358,000	16,000	4.4	12,000	4000
Total	1 096000	641,000	58	66,000	575,000

*HPV* human papillomavirus

This review aims to describe current therapeutic options for HPV-associated cancers, with an emphasis on therapeutic cancer vaccines currently being tested in clinical trials, and a particular focus on describing the efficacy and safety of the novel immunogenic compound axalimogene filolisbac (AXAL or ADXS11–001) in patients with HPV-positive cervical, head and neck, and anal cancers.

### Immunobiology of HPV

HPV belongs to a family of papillomaviruses that are composed of non-enveloped, double-stranded deoxyribonucleic acid (DNA) viruses able to infect the multilayer stratified tissue (eg, human epithelium). HPV is a sexually transmitted, circular virus encoding for 7 early (E1, E2, E4, E5, E6, E7, and E8) and 2 late, structural (L1 and L2) genes [4]. In cervical cancer, upon sexual transmission, HPV infects the basal epithelial cells of the cervical mucosa, leading to intracellular expression of low levels of viral proteins [5]. Viral DNA replicates following infection, and production of viral proteins is enhanced once HPV-infected cells leave the basal layer [6]. Chronic infection is maintained in approximately 10% of women because of the capacity of HPV to escape host immune surveillance [7]. The molecular mechanism accounting for persistent HPV infection and carcinogenesis involves the integration of viral DNA into the host genome, accompanied by deletion of both early and late HPV genes, namely E2, E4, E5, L1, and L2. The oncogenic potential of HPV is a result of two early viral proteins, E6 and E7. As a result of loss of the transcriptional regulator gene E2, these two oncoproteins are upregulated. The early viral protein E6 binds to the tumor suppressor gene p53, thereby inhibiting apoptosis of HPV-infected cells [8, 9]. The early viral protein E7 inhibits functionality of the tumor suppressor retinoblastoma product, thus allowing HPV to replicate in previously differentiated epithelial cells [9, 10]. Formation of complexes between these two viral proteins and the aforementioned tumor suppressor genes disturbs the normal cycle of cell regulation, causes genomic instability, and ultimately leads to neoplasia. A similar biomolecular process is the basis for development of other HPV-associated cancers.

## HPV-associated cancers and current therapies

### HPV-associated cancers and prevention of HPV infections

More than 100 HPV types have been identified to date [11]. Of these, the most frequently encountered high-risk HPV types, 16, 18, 31, and 45, are together responsible for approximately 80% of all cervical cancer cases [12–14]. HPV-16 and -18 have been identified as the two most prevalent high-risk HPV types and are accountable for approximately 62.6 and 15.7%, respectively, of cervical cancers [15]. Additionally, these two high-risk HPV types are responsible for 80–86% of vulvar and vaginal cancers, 89–95% of oropharyngeal cancers, 93% of anal cancers, and 63–80% of penile cancers [16].

Two prophylactic vaccines have been developed for prevention of HPV infection: Gardasil® (Merck and Co., Inc.), and Cervarix® (GlaxoSmithKline Biologicals). The quadrivalent vaccine Gardasil provides immunologic protection against infection with HPV-6, −11, −16, and −18 [17], whereas the bivalent vaccine Cervarix provides protection against infection with HPV-16 and -18 [18]. In addition, the nonavalent vaccine Gardasil 9 (Merck and Co., Inc.) has been demonstrated to protect against HPV-6, −11, −16, −18, −31, −33, −45, −52, and −58 [19]. However, despite recent advancements within the field of tumor immunology, no therapeutic vaccines for the treatment of HPV-associated cancers are currently available for general use in the clinical setting.

### Current therapeutic options for HPV-associated cancers

#### Cervical and vulvar cancers

Pre-invasive genital tract neoplasia includes cervical intraepithelial neoplasia (CIN), vaginal neoplasia, and vulvar intraepithelial neoplasia (VIN). Current treatment strategies for CIN include minimally invasive therapies, such as loop electrosurgical excision procedure or cryotherapy [20]. These strategies focus on eliminating the HPV-positive precancerous cells, while maintaining cervical integrity and fertility [21].

The estimated number of new cervical cancer cases raises to 528,000 each year [3], with 95% cases attributable to HPV [22], while from the 49,000 new cases of vulvar and vaginal cancers estimated, 41% were attributable to HPV [3]. Chances of survival are high when cervical cancer is identified at early stages. (International Federation of Gynecology and Obstetrics [FIGO] stages IA2–IB1). Treatment consists of conization, radical hysterectomy (preferred), radical trachelectomy (for selected patients), or radiation therapy. Locally advanced tumors are generally treated with concomitant chemoradiotherapy that includes a cisplatin-based regimen [23]. Patients with persistent, recurrent, or metastatic cervical cancer have poor survival and increased morbidity caused by renal failure and clinical deterioration. For these patients, the standard of care consists of platinum-based chemotherapy doublets, such as cisplatin and paclitaxel [24, 25], usually administered in combination with the humanized monoclonal antibody directed against vascular endothelial growth factor (bevacizumab) [26]. The main treatment modalities of vulvar cancer consist of surgery, for localized disease, or a combination of surgery and radiation (with or without chemotherapy) when nodal metastases are present [27, 28]. For patients presenting with vaginal cancers, three types of standard treatment are generally used: surgery (e.g., laser surgery, wide local excision, vaginectomy, total hysterectomy), external or internal radiation therapy [29], and systemic or regional chemotherapy [30].

#### Head and neck cancers

High-risk oncogenic HPVs represent major risk factors for development of head and neck cancers [31]. These are primarily tumors of the oropharynx, specifically the tonsil and base of tongue. An estimated 96,000 new cases of cancer of the oropharynx was recently reported, out of which 29,000 were attributable to HPV [3]. In the United States it has recently been approximated that HPV-related head and neck cancers incidence is likely to surpass that of cervical cancers by the year 2020 [32]. The primary viral etiology of these cancers is HPV-16; however, up to 9% may be caused by additional serotypes (e.g., HPV-35, HPV-18) [33]. Despite increasing awareness and improved viral detection methods, identification of active disease remains problematic [34]. Patients with early stage disease can be treated with single-modality therapy. Minimally invasive transoral robotic surgery is also being investigated [35]. The most commonly employed therapies for locally advanced head and neck cancers include cisplatin and concurrent radiation. Concurrent epidermal growth factor receptor antibody and radiation is an alternative. More recent treatments for patients with head and neck cancers include targeted therapies, such as the anti-programmed death protein 1 (PD-1) monoclonal antibody nivolumab. In a phase II trial performed in patients with recurrent or metastatic squamous cell carcinoma of the head and neck, the overall survival (OS) of patients treated with nivolumab was higher than OS following standard platinum chemotherapy (7.5 vs 5.1 months). The 6-month progression-free survival (PFS) rate and the response rate of patients treated with nivolumab were also higher than in patients receiving standard therapy (19.7% vs 9.9% and 13.3% vs 5.8%), while the occurrence of grade 3 or 4 treatment-related adverse events (TRAEs) was lower following nivolumab treatment (13.1% vs 35.1%) [36]. This proof-of-concept phase II trial not only demonstrated the superior efficacy of nivolumab in patients with recurrent or metastatic squamous cell carcinoma of the head and neck, but also underlined the superior safety profile of nivolumab in this patient population.

#### Anal and penile cancers

Annually, 24,000 anal and 11,000 penile cancer cases are reported worldwide, with approximately 21,000 and 6500 cases, respectively, associated with HPV [1]. In 2012, these numbers increased to 40,000 for anal cancer and 26,000 for penile cancer, out of which 35,000 and 26,000 respectively, were reported as attributable to HPV [3]. Anal intraepithelial neoplasia (AIN) is generally treated with minimally invasive methods, such as laser ablation or infrared coagulation [37]; excision is reserved for high-grade AIN cases. The standard of treatment for localized anal cancer is concurrent chemoradiotherapy, consisting of concurrent radiation, mitomycin C, and 5-fluorouracil [38, 39]. Metastatic anal cancer is not curable. While there is no standard-of-care chemotherapy, options can include platinum analogues, taxanes, or antimetabolites. The best options for localized penile cancer consist of surgical treatments (e.g., excision, microsurgery, laser surgery, circumcision) and radiation, used as an adjuvant to surgery. Current treatment options for recurrent or metastatic penile cancer includes taxanes, platinum analogues, and ifosfamide. [40, 41].

Despite the various treatment modalities currently available, survival of patients presenting with one of the aforementioned advanced, recurrent, or metastatic HPV-associated cancers remains poor.

#### Therapeutic HPV vaccines

In patients with HPV-associated cancers, the standard functionality of the innate and adaptive immune systems is altered and tolerance or suppression mechanisms develop, capable of blocking or reversing antitumor immune responses [42, 43]. Tolerance mechanisms interfere with various steps of the antigen-presentation process as well as with the antitumoral activity of cluster of differentiation (CD)4-positive and CD8-positive T cells, thus rendering them nonfunctional. Immune suppression mechanisms include development of suppressive immune cell populations (e.g., regulatory T cells [Tregs], myeloid-derived suppressor cells [MDSCs], tumor-associated macrophages) with protumoral activity. Overall, HPV-induced diseases are associated with a lack of HPV-specific antitumoral immune responses and an excess of immune-suppressive cellular and humoral protumoral responses. Therefore, the main goal of therapeutic vaccines is to induce or greatly improve HPV-specific T-cell based immunity by making use of the constitutively expressed tumor-specific antigens E6 and E7.

**Fig. 1:**
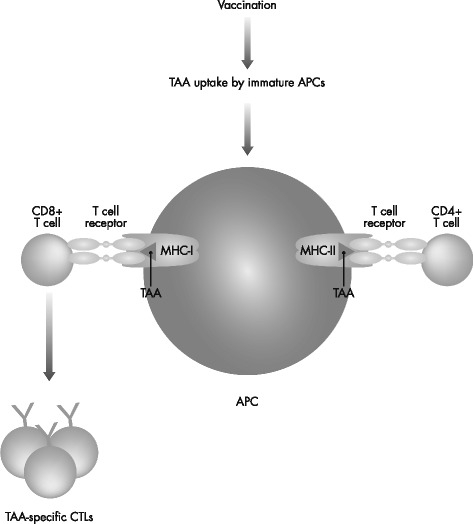
Schematic depiction of the general mechanism of action of therapeutic cancer vaccines. APC: antigen-presenting cell; CD: cluster of differentiation; CTL: cytotoxic T lymphocyte; MHC: major histocompatibility complex; TAA: tumor-associated antigen

Because of their reported efficacy, protein- and peptide-based vaccines are the most common forms of therapeutic HPV vaccines. Their mechanisms of action involve uptake of the peptide antigen and major histocompatibility complex molecules by dendritic cells, and cross-presentation to CD8-positive T cells (Fig. 1). A phase I study performed in patients with end-stage cervical cancer vaccinated with HPV-16 E6, alone or in combination with HPV-16 E7 overlapping long peptides, reported good vaccine tolerability and broad T-cell responses [44]. Several fusion protein-based vaccine formulations containing the oncogene E7 of the high-risk type HPV-16 have been tested in clinical trials in patients with high-grade AIN [45] or CIN [46, 47], as well as in patients with cervical cancer [48], and have demonstrated varying degrees of efficacy.

Nucleic acid and whole cell vaccines represent other potential immunotherapeutic strategies tested in clinical trials. A DNA vaccine containing E7 DNA fused with the heat shock protein 70 tested in patients with CIN grade 2/3 was reported to be safe, but induced only low-frequency E7-specific T-cell responses [49]. Similarly, autologous dendritic cells pulsed with HPV-16 or HPV-18 E7 recombinant proteins in patients with stage IB–IIA cervical cancer [50] or patients with late-stage disease [51] led to antigen-specific serologic responses of varying degrees; however, there was no sustained limitation on tumor burden. All of the aforementioned vaccine formulations were well tolerated and induced antigen-specific cell-mediated immunity to varying degrees. However, the rates of lesion regression observed within these studies were lower than 50%, with no direct correlation between clinical and immunologic responses reported. Recently, a phase II clinical trial was performed in patients with metastatic cervical cancer previously treated with chemotherapy or chemoradiotherapy. Following lymphocyte-depleting chemotherapy, patients received a single infusion of tumor-infiltrating T cells selected for HPV E6 and E7 reactivity. Objective tumor responses were observed in three of the nine patients enrolled: one patient presented with a three-month partial response, and two patients presented with complete responses that were ongoing at 22 and 15 months after treatment, respectively. This proof-of-concept study demonstrated durable, complete regression of metastatic cervical cancer following a single infusion of HPV-specific tumor-infiltrating T cells [52].

Another promising option for therapeutic vaccination is bacterial or viral vector-based vaccines (e.g., sindbis virus, equine encephalitis virus, or adenovirus). Thus far, the efficacy of some viral vector vaccines has been reported in preclinical models, while others have been tested in clinical trials. A recombinant vaccinia virus expressing the E2 protein of HPV-16 or HPV-18 led to complete lesion regression in CIN grade 2/3 patients [53], whereas a vaccine expressing a fusion protein of E6 and E7 caused therapeutic effects in patients with VIN in phase I/II clinical trials [54].

Nevertheless, survival outcomes of patients with HPV-associated cancers treated with the aforementioned vaccine combinations need to be greatly enhanced.

#### Axalimogene filolisbac (AXAL or ADXS11–001)

The bacterial vector most commonly used as an immunotherapeutic vaccine base is *Listeria monocytogenes* (*Lm*)*,* because of its immunologic advantages. *Lm* is a gram-positive intracellular bacterium capable of escaping from the host cell phagosomes into the cytoplasm, thereby infecting host cells. Following infection of host cells, *Lm* has the ability to activate both the innate (neutrophils and macrophages) [55] and adaptive (CD4-positive and CD8-positive T cells) [56] immune responses. In cancer immunotherapy, *Lm* has been successfully used as a delivery vector for tumor-specific antigens.

*Lm*-listeriolysin O (LLO) immunotherapies have been reported to present with multiple simultaneous mechanisms of action that contribute to generation of a therapeutic response, enabled by their capacity to efficiently stimulate both innate and adaptive immune responses [57]. Upon administration, *Lm*-LLO immunotherapies have been shown to infect antigen-presenting cells, thereby initiating the process of antigen cross-presentation. This effect propagates to both arms of the adaptive immune system, leading to generation of activated CD4-positive and CD8-positive T cells. Additionally, *Lm*-LLO immunotherapies selectively reduce levels of intratumoral, but not splenic, Tregs and MDSCs, and present the capacity to induce maturation of immune cells to fully differentiated effector cells devoid of protumoral activity. Other advantages of *Lm*-LLO immunotherapies are their lack of induction of neutralizing antibodies and their capacity to facilitate chemotaxis of activated immune cells. Interestingly, *Lm*-LLO immunotherapies also stimulate robust immune memory responses; correlates of immune memory to *Lm* have been reported to develop just 5 h after exposure [58]. In various models, *Lm*-LLO immunotherapies have been shown to also induce therapeutic changes in the ratio of CD8-positive tumor-infiltrating lymphocytes to Tregs [59].

Because of the aforementioned promising results, one such *Lm*-LLO immunotherapy was used for development of AXAL, a novel immunotherapeutic agent for treatment of cervical cancer and other HPV-associated diseases. AXAL is based on the live, irreversibly attenuated *Lm* fused to the nonhemolytic fragment of LLO, and has been developed to secrete the *Lm*-LLO-E7 fusion protein targeting HPV-positive tumors [60]. AXAL is also bioengineered to be deficient of virulence-related transcription factors, such as peptide-chain release factor A [61, 62], induces antitumor T-cell immunity, and reduces tumor immune tolerance.

### AXAL in clinical trials

Presently, AXAL is being evaluated in several clinical trials of patients with various HPV-associated tumors (Table 2) [61, 63–71]. Thus far, AXAL has most extensively been evaluated in cervical cancer, with various recently finalized or currently ongoing clinical trials having enrolled patients with cervical cancer at different stages.

**Table 2:** Overview of AXAL in clinical studies (safety and efficacy)

Cancer type	Cancer stage	Investigator	Study phase (stage; NCT)	Mono−/Multi-therapy	Dosing regimen	Estimated enrollment	Efficacy	Safety (most frequent AEs)
Cervical cancer	Advanced	Maciag PC	I (60)	AXAL alone	Dose escalation • 1 × 10^9^ CFU • 3.3 × 10^9^ CFU • 1 × 10^10^ CFU	15	• Possible PR: 1 patient • SD: 7 patients • Progression of disease: 5 patients	• Pyrexia (100%) • Vomiting (60%) • Musculoskeletal pain (57%) • Chills; headache and anemia (53%) • Nausea and tachycardia (47%) DLT = 1 × 10^10^ CFU
	Persistent/recurrent/metastatic	GOG (Huh WK)	II (stage 1; NCT01266460) (62)	AXAL alone	1 × 10^9^ CFU	67	• 12-month OS: 38.5% • Median PFS: 3.1 mo • Median OS: 7.7 mo • PR: 1 patient • SD: 9 patients	Drug-related AEs (38% of all AEs) • Vomiting • Chills • Fatigue • Fever
	Persistent/recurrent/metastatic	Ghamande SA	I-II (TiP; NCT02164461) (63)	AXAL alone	Dose escalation • 5 × 10^9^ CFU • 1 × 10^10^ CFU	25	Pending	Treatment-related AEs (>3 patients) • Chills • Vomiting • Hypotension • Tachycardia • Fever • Nausea
	Recurrent/refractory	Petit R	II (CTRI/2010/091/001232) (64)	AXAL ± cisplatin	1 × 10^9^ CFU + 40 mg/m^2^	110	• 12-mo OS: 36% • 18-mo survival: 28% • Response rate: 11% (6 CRs; 6 PRs) • SD: 35 patients	• 79% of AEs: mild or moderate and unrelated to study drug
	High-risk locally advanced	GOG (Herzog TJ)	III (TiP; NCT02853604) (65)	AXAL alone	1 × 10^9^ CFU	450	Pending	Pending
Head and neck cancer	Persistent/recurrent/metastatic	Cohen EW	I/II (TiP; NCT02291055) (66)	AXAL ± MEDI4736	Phase I: 1 × 10^9^ CFU + 3 mg/kg (3 + 3 design for MEDI4736 dose escalation) Phase II: 1 × 10^9^ CFU + 10 mg/kg	66	Pending	Pending
	Previously untreated, surgically resectable, stage II–IV patients (oropharyngeal cancer)	Miles B and Sikora A	II (NCT02002182) (67)	AXAL + transoral robotic surgery	1 × 10^9^ CFU	30 (present time: 8/9 vaccinated patients; 10 observational group patients)	• Increased Ag-specific IFN-γ (5/8) or TNF-α (78) responses at 3/5 time points (other 2 time points pending) • Intratumoral expression of CD8 (4/8), PD-1 (6/8)	Pending
	Oropharyngeal cancer	Jones TM	I (NCT01598792) (68)	AXAL alone	Dose escalation • 3.3 × 10^8^ CFU • 1 × 10^9^ CFU • 3.3 × 10^9^ CFU	36	Pending	Pending
Anal cancer	Locally advanced	Safran H	I/II (TiP; NCT01671488) (69)	AXAL ± chemo-radiation (mitomycin, 5-fluorouracil, IMRT)	1 × 10^9^ CFU	25	Pending	Pending
	Persistent/recurrent, locoregional/metastatic anorectal canal	Fakih M	II (stage 2 TiP; NCT02399813) (70)	AXAL alone	1 × 10^9^ CFU	Stage 1 • 31 patients Stage 2 • 24 patients	Pending	Pending

*Ag* antigen, *BID* bi-daily, *CR* complete response, *CTRI* Clinical Trials Registry – India, *DLT* dose-limiting toxicity, *IMRT* intensity-modulated radiation therapy, *NCT* National Clinical Trial, *PR* partial response, *SAE* serious adverse event, *TiP* trial in progress

#### AXAL in patients with cervical cancer

In 2009, Maciag et al. [61] published the first phase I clinical trial of AXAL, in which safety and efficacy were assessed in 15 patients with advanced cervical cancer whose disease presented no improvements following traditional therapies. Women with a history of listeriosis were excluded from this study. Doses of AXAL escalating from 1 × 10^9^ colony-forming units (CFU) to 3.3 × 10^9^ CFU and then 1 × 10^10^ CFU were administered to groups of five patients every 21 days for a total of two intravenous doses. Patients also received prophylactic antibiotics. Dose-limiting toxicity was achieved at the highest dose of 1 × 10^10^ CFU, with three of five patients developing hemodynamic instability and subsequently treated with medical interventions. All 15 patients reported at least one AE, as classified by the Common Terminology Criteria for Adverse Events version 3.0 [72]. The most common AEs reported by more than 50% of patients during the study were pyrexia (100%), vomiting (60%), musculoskeletal pain (57%), and chills, headache, and anemia (53%). Blood, urine, and feces analyses revealed the transient presence of *Lm*-LLO-E7 in only one patient receiving 1 × 10^9^ CFU. Within the follow-up period of the study, two deaths were recorded. One death was caused by disease progression, while the second occurred in the setting of renal failure, followed by metabolic acidosis and cardiac arrest. Both were deemed unrelated to AXAL [61]. Overall, the results of this study showed an acceptable safety profile for AXAL at the dose of 1 × 10^9^ CFU.

Considering the acceptable safety profile of AXAL observed in the study of Maciag et al., a single-arm, two-stage, phase II clinical trial is being conducted in patients with squamous or nonsquamous persistent, recurrent, metastatic cervical cancer that has progressed after systemic chemotherapy. In a Gynecologic Oncology Group (GOG) study, a total of 67 patients was estimated for enrolment at study initiation, with a target of 27 patients enrolled in the first study stage [63] (Table 2) [61, 63–71]. Eligible patients are 18 years of age or older, have a GOG performance status of 0 or 1, have measurable disease (Response Evaluation Criteria in Solid Tumors [RECIST] version 1.1), and have received one or more prior lines of systemic-dose chemotherapy (bevacizumab permitted) for squamous or non-squamous persistent or recurrent metastatic cervical cancer not amenable to curative therapy. In this study, AXAL safety and tolerability, as well as 12-month OS rates, were evaluated following administration of three doses of AXAL at 1 × 10^9^ CFU every 28 days. Secondary endpoints were PFS, OS, and objective response (OR). To prevent development of the most common AEs reported in the study by Maciag et al., nonsteroidal anti-inflammatory agents were administered prophylactically. In total, 26 of the 29 patients who were enrolled in stage 1 received treatment. Safety analyses indicated that all treated patients experienced at least one AE: 91% were grade 1–2, and 38% were drug related, with nausea, vomiting, chills, fatigue, and fever the most common. The 38.5% (10 patients) 12-month OS rate observed in stage 1 of the study suggests that AXAL is an active agent with a net survival benefit for patients with squamous or nonsquamous persistent or recurrent metastatic cervical cancer. Median PFS was 3.1 months and median OS was 7.7 months. Preliminary evaluation of OR showed that one patient presented with unconfirmed partial response and nine patients presented with stable disease. Post-hoc efficacy analysis of the 18 patients who received all three per-protocol doses of AXAL showed a median OS longer than 1 year, and a 12-months OS rate of 55.6%, thus confirming the survival benefit offered by AXAL.

Another phase I, open-label, dose-escalation clinical trial being performed in patients with persistent, recurrent, or metastatic cervical squamous carcinoma or adenocarcinoma aims to evaluate the safety and tolerability of higher doses of AXAL, as well as tumor response, PFS, and correlative immunologic parameters [64] (Table 2) [61, 63–71]. Patients enrolled in this study had measurable disease (RECIST version 1.1) with documented disease progression on or intolerance to prior therapy, and had an Eastern Cooperative Oncology Group (ECOG) performance status of 0 or 1. Overall, 10 of 25 patients were enrolled, and nine of 10 patients were treated with AXAL every 3 weeks during a 12-week treatment cycle (six patients received 5 × 10^9^ CFU and three patients received 1 × 10^10^ CFU). The primary endpoint of this study was AXAL safety and tolerability, with the recommended phase II dose (RP2D) selected based on a dose-limiting toxicity rate lower than 33%. Secondary objectives included evaluation of tumor response and PFS. All treated patients experienced at minimum one AE, of which 75% were TRAEs (eight of nine patients): 99% were grade 1–2, and the most common TRAEs occurring in three or more patients were chills, vomiting, hypotension, tachycardia, fever, and nausea. Only one grade 3 (hypotension) and no grade 4–5 TRAEs were reported. Analysis of tumor response and PFS, as well as correlative immunologic studies, is ongoing to assess if treatment intensity has an impact on the antitumor activity of AXAL. Lastly, another phase III clinical trial of AXAL (AIM2CERV) administered as adjuvant immunotherapy in patients with high-risk, locally advanced cervical cancer following chemoradiation was opened for recruitment in September 2016 [65] (Table 2) [61, 63–71].

Given the observed safety and efficacy of AXAL when administered alone in patients with cervical cancer, combinatorial therapies containing AXAL were also assessed in clinical trials. The efficacy and safety of AXAL, administered with or without cisplatin, was evaluated in a phase II trial performed in India that enrolled 110 patients with recurrent or progressive invasive cervical cancer unresponsive to primary therapy [65] (Table 2) [61, 63–71]. Eligible patients were 18 years of age or older, had documented recurrent or progressing invasive cervical cancer, presented with measurable disease with at least one target lesion, and had ECOG performance status 2 or lower. Patients were randomized to AXAL alone (one cycle of three doses at 1 × 10^9^ CFU administered every 4 weeks) or AXAL plus cisplatin (one preliminary AXAL dose followed by five weekly cisplatin treatments at the dose of 40 mg/m^2^, followed by one AXAL cycle). The primary endpoint was OS; secondary endpoints were OR rates, PFS, and safety. Among the 109 patients who received treatment, AXAL was well tolerated; 79% of AEs were mild or moderate and unrelated to study drug. OS was found to be similar between the two treatment arms (median OS AXAL: 8.40 months; AXAL with cisplatin: 8.77 months). Furthermore, 22% of the treated patients alive at more than 18 months following AXAL therapy were deemed long-term survivors. No significant differences between the OR rates, disease control rates, duration of response, or PFS were observed between the two treatment groups.

#### AXAL in patients with head and neck cancer

In addition to the clinical trials performed in patients with cervical cancer, AXAL is also being investigated in other types of HPV-positive cancers, such as head and neck and anorectal cancer. Three phase I/II clinical trials in patients with head and neck cancer and two phase I/II clinical trials in patients with anorectal cancer are currently ongoing. The phase I/II randomized two-stage study by Cohen et al. is being performed in patients with recurrent, HPV-positive squamous cell carcinoma of the head and neck or cervix [67] (Table 2) [61, 63–71]. In phase I of this study, safety and efficacy of 1 × 10^9^ CFU of AXAL administered every 4 weeks in combination with the PD-1 inhibitor durvalumab (MEDI-4736), used at escalating doses (dose level 1: 3 mg/kg; dose level 2: 10 mg/kg) administered every 2 weeks, will be assessed, and the RP2D of the combination therapy will be determined in up to 18 patients. In phase II of the study, 48 patients will be randomized to receive AXAL (1 × 10^9^ CFU), durvalumab, or both, at the pre-established RP2D. The study allows patients to receive treatment for up to 1 year or, alternatively, discontinue treatment due to disease progression or unacceptable toxicity, and is currently ongoing.

Another clinical study of AXAL in patients with previously untreated, surgically resectable, stage II–IV oropharyngeal cancer is the “window of opportunity” phase II, two-stage trial that was initiated in 2014 [68] (Table 2) [61, 63–71]. Patients received AXAL at 1 × 10^9^ CFU (two doses over 5 weeks, on the first and fifteenth days of treatment, respectively), prior to standard-of-care transoral robotic surgery. Of the eight of nine enrolled patients who completed study treatment, five patients presented with increased peripheral blood antigen-specific interferon gamma (IFN-γ) or tumor necrosis factor alpha (TNF-α) responses before treatment, on the day of surgery, and 5 weeks postsurgery. Additionally, intratumoral increases in posttreatment expression of CD8-positive T cells and PD-1 were recorded in four of eight and six of eight patients, respectively. These results are promising, as they indicate that effects of AXAL are not limited to the generation of robust antitumoral immune responses, but are also extended to the tumor microenvironment. Another phase I clinical trial evaluating the safety of escalating AXAL doses (from 3.3 × 10^8^ to 3.3 × 10^9^ CFU) in oropharyngeal cancer is also in progress [63] (Table 2) [61, 63–71].

#### AXAL in patients with anal cancer

In view of the reported efficacy of AXAL in cervical cancer and available immune response data in head and neck cancers, clinical trials aiming to evaluate this immunotherapeutic compound in HPV-positive anal cancers have recently been initiated. The phase I/II study by Safran et al. aims to assess safety and efficacy of AXAL when combined with intensity-modulated radiation therapy, mitomycin, and 5-fluorouracil for treatment of patients with anal cancer [70] (Table 2) [61, 63–71]. The phase II, two-stage study by Fakih et al. aims to assess the efficacy and safety of AXAL monotherapy (administered intravenously at a dose of 1 × 10^9^ CFU every 3 weeks during nine-week treatment cycles) in patients with persistent, recurrent, locoregional, or metastatic squamous cell cancer of the anus. [71] (Table 2) [10, 63–71]. Both of these trials are ongoing, and results are expected to be made available in 2017.

### Advantages of treatment with AXAL and future perspectives

**Fig. 2:**
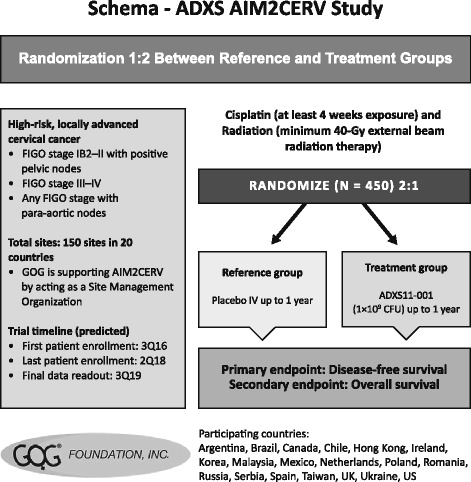
AXAL planned phase III study. AXAL: axalimogene filolisbac; FIGO: International Federation of Gynecology and Obstetrics; GOG: Gynecologic Oncology Group; Q: quarter; UK: United Kingdom; US: United States

AXAL seems to embody the main characteristics of a successful HPV therapeutic vaccine for patients with various HPV-associated cancers, both in terms of efficacy (i.e., capacity to engage both innate and adaptive immunity by promoting inflammation and inducing high numbers of antigen-specific cytotoxic T lymphocytes) and safety (i.e., reported tolerability). Additional benefits include the ability of AXAL to dampen intratumoral immune tolerance by reducing numbers and functionality of Tregs and MDSCs, as well as reducing secretion of immunosuppressive cytokines (e.g., interleukin-10 and transforming growth factor beta), both features of an immune response that would ordinarily contribute to a protumor microenvironment. Another advantage of AXAL immunotherapy is exemplified by its delivery vector, *Lm,* an attenuated bacterial vector characterized by lack of virulence yet retaining its adjuvant properties. Moreover, given the conserved nature of the E7 early gene in HPV, AXAL is expected to be effective irrespective of the HPV serotype associated with individual cases, which is particularly important in heterogeneous patient populations such as those with head and neck cancer. A further advantageous aspect of AXAL therapy is its potential to provide long-term immune modulation, an aspect of treatment that is currently lacking. In the case of HPV-associated oropharyngeal squamous cell carcinomas, which are known to involve longer survival and later recurrence compared to other head and neck cancers [72], the use of AXAL to bolster the antitumor response in conjunction with disease surveillance is an exciting yet currently unexplored option for preventing late recurrence in this setting. Potentially, treatment with AXAL could have an immediate impact improving patients’ perception of care, which is often negatively affected by delays in scheduling surgery or radiotherapy. The effective and safe administration of AXAL in the weeks prior to definitive treatment has been indicated to be plausible in the window of opportunity study [68]. Another study that may have significant bearing on future strategies for AXAL use is AIM2CERV, the randomized phase III study of AXAL use following chemoradiation in patients with high-risk, locally advanced cervical cancer [66] (Fig. 2). In this setting, there is a clear unmet need, as patients have a 50% probability of disease recurrence or death following cisplatin-based chemoradiation plus brachytherapy. AIM2CERV will evaluate disease-free and OS as its endpoints; study enrollment is currently ongoing. Based on the studies described above and the windows of opportunity naturally occurring in different therapeutic scenarios, future directions of treatment with AXAL will most likely include administration in oropharyngeal squamous cell carcinomas and high-risk locally advanced cervical cancer, in combination with standard treatment options or alone.

## Conclusions

Considering the rising incidence of various HPV-associated cancers within the last years [73, 74], as well as HPV infection being more recently established as the principal cause of increased incidence in head and neck cancers [74], novel therapeutic options for HPV-associated cancers are stringently necessary. Novel therapeutic options for HPV-associated cancers include the use of vaccines based on DNA, peptides, or viral vectors. Delivery of HPV antigens using viral vectors, such as in the case of AXAL, has several advantages: in contrast to peptide immunization, CTL epitopes can be processed/presented naturally and delivered more effectively to target cells; in contrast to DNA immunization, the efficiency of introducing heterologous genes in target cells can be enhanced. While the exact costs for treatment with AXAL are not yet completely elucidated, given the long-term beneficial effects reported upon its administration to patients with HPV-positive tumors, it can be hypothesized that AXAL treatment will be cost-effective. Furthermore, considering the relatively straightforward method of AXAL production, the costs of a full course of AXAL are hypothesized to be far inferior to those of Sipuleucel-T treatment, for which a full course rises to $98,780 [75]. A more specific cost-estimate of the treatment will hopefully be possible in the near future, especially since treatment with AXAL is expected to be available within the next 5 years or earlier. In this regard, patients with high-risk locally advanced cervical cancer represent the ideal target patients for treatment with AXAL, also based on the promising results obtained so far in this patient population.

Although there is evidence in the literature demonstrating a growing incidence of decreased vaccine acceptance and hesitancy of usage [76], the various ongoing clinical trials with AXAL indicate its potential for widespread use across various types of HPV-associated cancers, thus placing AXAL among the promising immunotherapeutic tools of the future.

## Abbreviations

Ag: Antigen
AIN: Anal intraepithelial neoplasia
AXAL: Axalimogene filolisbac
BID: bi-daily
CD: Cluster of differentiation
CFU: Colony-forming unit
CIN: Cervical intraepithelial neoplasia
CR: Complete response
CTRI: Clinical Trials Registry – India
DLT: Dose-limiting toxicity
DNA: Deoxyribonucleic acid
ECOG: Eastern Cooperative Oncology Group
FIGO: Federation of Gynecology and Obstetrics
GOG: Gynecologic Oncology Group
HPV: Human papillomavirus
IFN-γ: Interferon gamma
IMRT: Intensity-modulated radiation therapy
LLO: Listeriolysin O
*Lm*: *Listeria monocytogenes*
MDSCs: Myeloid-derived suppressor cells
NCT: National Clinical Trial
OR: Objective response
OS: Overall survival
PD-1: Programmed death protein 1
PFS: Progression-free survival
PR: Partial response
Q: Quarter
RECIST: Response Evaluation Criteria In Solid Tumors
RP2D: Recommended phase II dose
TiP: Trial in progress
TNF-α: Tumor necrosis factor alpha
TRAEs: Treatment-related adverse events
Tregs: Regulatory T cells
UK: United Kingdom
US: United States
VIN: Vulvar intraepithelial neoplasia
